# The Effects of Resveratrol on Inflammation and Oxidative Stress in a Rat Model of Chronic Obstructive Pulmonary Disease

**DOI:** 10.3390/molecules22091529

**Published:** 2017-09-12

**Authors:** Xiao-Li Wang, Ting Li, Ji-Hong Li, Shu-Ying Miao, Xian-Zhong Xiao

**Affiliations:** 1 Department of Pathophysiology, Xiangya School of Medicine, Central South University, Changsha 410000, Hunan, China; wangxiaolif@163.com (X.-L.W.); cdx8080@163.com (T.L.); 18673176715@163.com (S.-Y.M.); 2 Department of Pathology, Medical College of Jishou University, Jishou 416000, Hunan, China; ljh200500@163.com

**Keywords:** cigarette smoke, lipopolysaccharide, resveratrol, SIRT1, PGC-1α, oxidative stress, COPD

## Abstract

Oxidative stress and inflammation are hypothesized to contribute to the pathogenesis of chronic obstructive pulmonary disease (COPD). Resveratrol (trans-3,5,4′-trihydroxystilbene) is known for its antioxidant and anti-inflammatory properties. The study aimed to investigate the effects of resveratrol in a rat model with COPD on the regulation of oxidative stress and inflammation via the activation of Sirtuin1 (SIRTl) and proliferator-activated receptor-γ coactivator-1α (PGC-1α). Thirty Wistar rats were randomly divided into three groups: control group, COPD group and resveratrol intervention group. The COPD model was established by instilling with lipopolysaccharide (LPS) and challenging with cigarette smoke (CS). The levels of interleukin-6 (IL-6) and interleukin-8 (IL-8) in serum were measured. The levels of malondialdehyde (MDA) and the activity of superoxide dismutase (SOD) were measured. The expression levels of SIRT1 and PGC-1α in the lung tissues were examined by immunohistochemistry as well as real-time reverse transcriptase polymerase chain reaction (real-time RT-PCR) and western blotting analysis. After the treatment with resveratrol (50 mg/kg), compared with the COPD group, alleviation of inflammation and reconstruction in the small airways of the lungs were seen. Resveratrol might be correlated not only with the lower level of MDA and the higher activity of SOD, but also with the upregulation of SIRT1 and PGC-1α expression. Resveratrol treatment decreased serum levels of IL-6 and IL-8. Our findings indicate that resveratrol had a therapeutic effect in our rat COPD model, which is related to the inhibition of oxidative stress and inflammatory response. The mechanism may be related to the activation and upgrading of the SIRT1/PGC-1α signaling pathways. Thus resveratrol might be a therapeutic modality in COPD.

## 1. Introduction

It is reported that chronic obstructive pulmonary disease (COPD) is one of the leading causes of death worldwide, and mortality rates will become the third leading cause of death by 2030 [1]. Oxidative stress, protease–antiprotease imbalance and inflammation are important in the pathogenesis of COPD [2]. Lipopolysaccharide (LPS) and cigarette smoke (CS) have become the preferred stimuli for researching COPD [3]. CS is a mixture of oxidant radicals and different chemical compounds, which cause oxidative stress in the lungs. LPS is an endotoxin of gram negative bacteria, which can induce lung injury. CS and LPS were combined to replicate severe inflammation and oxidative stress in rat lungs in this study. The role of cell signaling pathway dysfunction and oxidative stress in COPD were recognized.

CS augments the production of reactive oxygen species (ROS) and numerous pro-inflammatory cytokines such as IL-6 and IL-8 [4].The lipid peroxidation product malondialdehyde (MDA) is the most commonly measured indicator of oxidative damage to membrane lipids [5]. Superoxide dismutase (SOD) eliminates superoxide and reduces oxidative stress and tissue damage. There is believed to be a need for a new therapy for candidates exhibiting anti-oxidant and anti-inflammatory properties for COPD. SIRT1 is a well-known longevity gene, which regulates stress resistance and inflammation by deacetylation of intracellular signaling molecules and histones [6], which probably acts via different mechanisms to regulate age-related changes including increasing mitochondriogenesis via modulating PGC-1α deacetylation [7].

Resveratrol is a phytoalexin found in the skin and seeds of grapes, recent studies have demonstrated the role of resveratrol in lung injuries induced by different xenobiotics via the antioxidant and anti-inflammatory pathway [5]. Resveratrol restored ovarian function through diminishing ovarian inflammation, predominantly via upregulation of SIRT1 expression leading to the inhibition of inflammatory cytokines [8]. Resveratrol could also inhibit COPD-associated cytokines/chemokines such as IL-6 and IL-8 from releasing from the human airway smooth muscle cells (HASMCs) through the activation of SIRT1 [9].

However, to our knowledge, it is unknown whether resveratrol reduces inflammation and oxidative stress in the lungs of COPD via the SIRT1/PGC-1α signaling pathway. This study was designed to evaluate the effects of resveratrol on COPD in rats. It was hypothesized that upregulation of SIRT1/PGC-1α expression might represent essential regulatory mechanisms implicated in the effects of resveratrol.

## 2. Results

### 2.1. Resveratrol Reduced Lung Inflammatory Response

**Figure 1:**
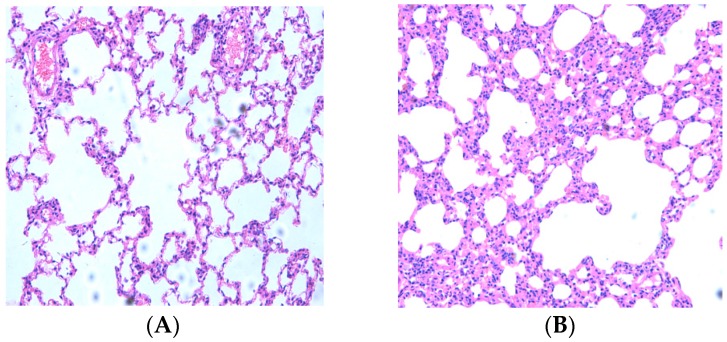

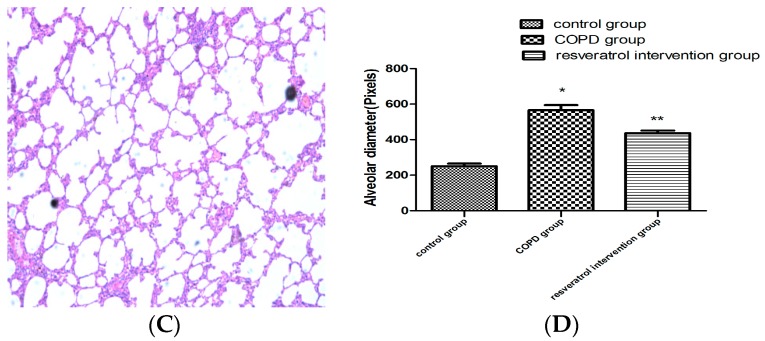
Micrographs of lung histopathology. Histological changes of lung segment stained with HE after resveratrol treatment. (**A**) control group (n = 10): No abnormalities were seen; (**B**) COPD group (n = 10): Pulmonary tissue showed a severe inflammatory response with visible increases in inflammatory cells; (**C**) resveratrol group (n = 10): Inflammation lessened after resveratrol therapy. The number of inflammatory cells decreased. (Magnification, ×200); (**D**) Alveolar diameter was evaluated. Values represent mean ± SEM, * *p* < 0.01, vs. Control group (n = 10); ** *p* < 0.01, vs. COPD group (n = 10).

First, the effects of CS and LPS on the destruction of the alveolar structure were evaluated. Histopathological examination of hematoxylin and eosin (HE) stained lung sections showed severe granulocyte infiltration of bronchial epithelium, and necrosis of bronchial mucosal epithelium cells. The alveolar diameter was examined by using Image-Pro Plus^®^ 6.0 software (Media Cybernetics, Inc, Rockville, MD, USA). Compared to control rats, COPD rats showed a severe inflammatory response with visible increases in inflammatory cells, with most of them being neutrophils and lymphocyte and alveolar macrophages. The alveolar sacs and alveolar spaces were enlarged, and the alveolar walls were thickened (Figure 1A,B). Treatment with resveratrol offered some reduction in the extent of cellular infiltration, the number of inflammatory cells decreased and the alveolar sacs and alveolar spaces were relieved when compared with the COPD group (Figure 1C). Resveratrol protected against the CS- and LPS-induced pathologic destruction and inflammatory infiltration of the lungs.

### 2.2. Resveratrol Decreased the Number of Inflammatory Cells inBronchial Alveolar Lavage Fluid (BALF)

**Figure 2:**
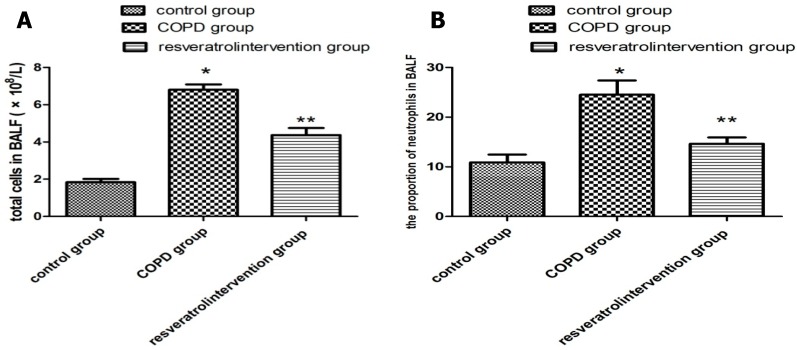
Resveratrol suppressed the recruitment of inflammatory cells into BALF in COPD rats. The numbers of total cells (**A**) the proportion of neutrophils (**B**) in BALF: the COPD with resveratrol group were significantly decreased compared with the COPD model group. Data are mean ± SD (n = 10). * *p* < 0.05 versus the control group, ** *p* < 0.05 versus the COPD model group.

In order to assess whether resveratrol influenced inflammation in lungs, we analyzed the numbers of inflammatory cells in BALF. As presented in Figure 2, the LPS and CS challenge resulted in increased numbers of the total leukocyte count and neutrophils in BALF compared with the control group. Compared with the COPD group, resveratrol treatment led to a significant reduction in the total cell count, and the relative count of neutrophils. (*p* < 0.05, Figure 2A,B).

### 2.3. Resveratrol Decreased the Expression of IL-6 and IL-8 in Serums

**Figure 3:**
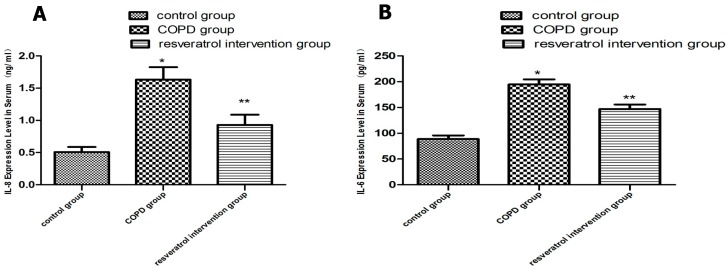
Effects of resveratrol on inflammation in COPD rats. Enzyme–linked immunosorbent assay (ELISA) was performed to detect levels of IL-6 and IL-8 in serums (**A**,**B**) resveratrol can reduce the levels of IL-6 and IL-8 (n = 10). * *p* < 0.05, vs. Control group (n = 10); ** *p* < 0.05, vs. COPD group (n = 10).

Next, to test whether resveratrol modulated the inflammatory process in COPD, the expression of IL-6 and IL-8 in serums was evaluated. Inflammatory cells release various cytokines including IL-6 and IL-8, which play an important role in inflammatory response. At the end of thirty days, serum levels of IL-6 and IL-8 were significantly higher in the COPD group compared to the control group (*p* < 0.05), while those in the resveratrol group were significantly lower than those in the COPD group (*p* < 0.05, Figure 3A,B). The results showed that resveratrol inhibited cytokine expression (IL-6 and IL-8) in COPD. These findings indicate that resveratrol can alleviate COPD inflammation by reducing interleukin levels.

### 2.4. Resveratrol Decreased the Activity of MDA and Increased the Activity of SOD

**Figure 4:**
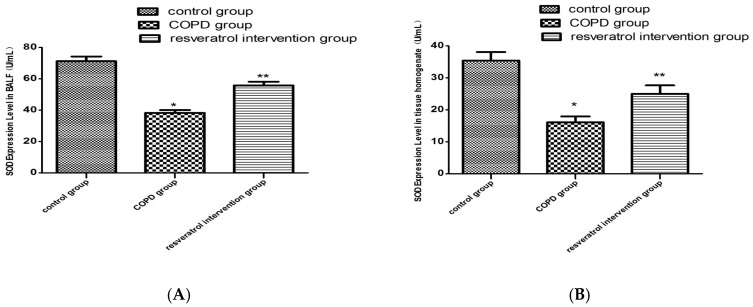

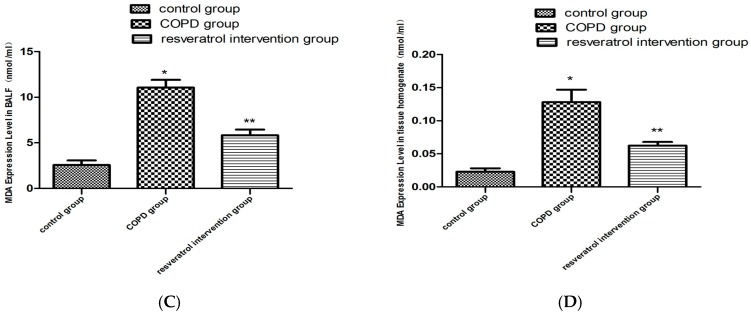
Effects of resveratrol on oxidative stress in COPD rats. SOD and MDA in both BALF and pulmonary tissue omogenate were measured as described in the methods. Resveratrol increases the activity of SOD (**B**) and decreases the concentration of MDA (**D**) in lung tissues of the resveratrol group (n = 10), the same result as in BALF (**A** and **C**). * *p* < 0.01, vs. Control group (n = 10); ** *p* < 0.01, vs. COPD group (n = 10).

The total MDA levels and SOD activities in the lung tissues and BALF were investigated to evaluate the effect of resveratrol on changes in oxidant–antioxidant systems. As shown in Figure 4A, the levels of SOD in the COPD group were significantly lower than those in the control group (*p* < 0.01). While after resveratrol treatment the SOD activity was still lower than that in the control group, it was significantly higher than that in the COPD group (Figure 4B). Contrary to the antioxidant enzyme SOD, MDA is one of the major oxidative damage markers. The MDA content of the COPD group markedly increased in the lung tissues (*p* < 0.05), but in the resveratrol treatment group it obviously decreased when compared with the model group (*p* < 0.01, Figure 4C,D). Pretreatment with resveratrol significantly decreased MDA levels and increased SOD activity. These findings indicate that resveratrol can alleviate increased oxidative stress induced in the COPD condition.

### 2.5. Effects of Resveratrolon the Expression of SIRT1 and PGC-1α in Lung Tissues

**Figure 5:**
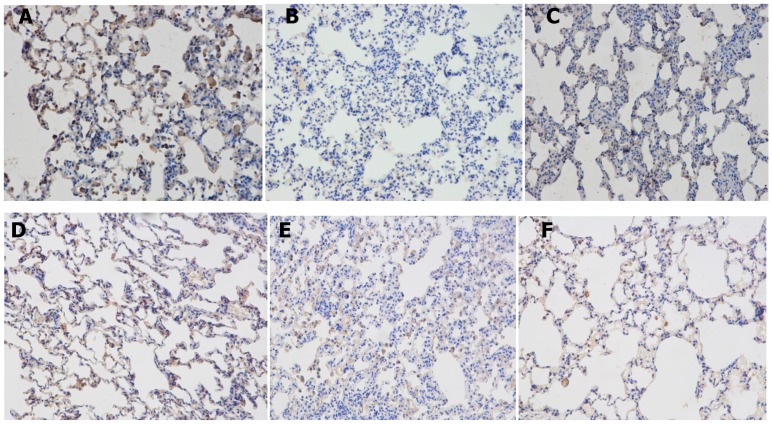
Expressions of SIRT1 and PGC-1α in lung tissues in rats. (**A**) Expression of SIRT1 in lung tissues in the control group; (n = 10) (**B**) SIRT1 protein in the lung in the COPD group; (n = 10) (**C**) SIRT1 protein in the lung in the resveratrol intervention group (n = 10) (Magnification, ×200) (**D**) Expression of PGC-1α in lung tissues in the control group; (n = 10) (**E**) PGC-1α protein in the lung in the COPD group; (n = 10) (**F**) PGC-1α protein in the lung in the resveratrol intervention group. (n = 10) (Magnification, ×200).

**Figure 6:**
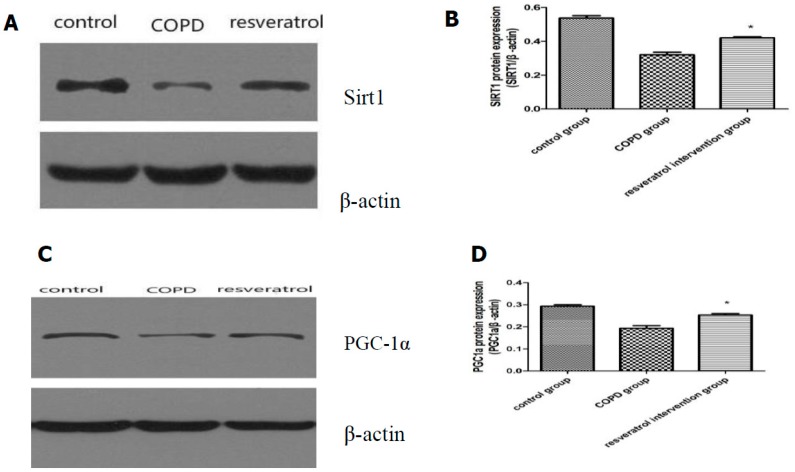
SIRT1 and PGC-1α protein expression: (**A**) representative western blots of SIRT1 and (**B**) the results of the quantitative densitometric analysis. SIRT1 protein decreased in the lungs of COPD rats (n = 10). Lungs of resveratrol rats (n = 10) showed higher levels of SIRT1; (**C**) Representative western blots of PGC-1α and (**D**) the results of the quantitative densitometric analysis. PGC-1α protein decreased in the lungs of COPD rats. Lungs of resveratrol rats (n = 10) showed higher levels of PGC-1α. * significant (*p* < 0.05) vs. with COPD rats (n = 10).

In order to assess whether resveratrol enhances the expression and activity of SIRT1 and PGC-1α, we analyzed the expression of SIRT1 and PGC-1α in the lung tissues, then we also analyzed the transcription of the SIRT1 and PGC-1α gene by real-time PCR and the expression of SIRT1 and PGC-1α protein by western blotting analysis. Using immunohistochemical staining (Figure 5B–F), SIRT1 was observed in the nucleus and PGC-1α was observed mainly in the cytoplasm. The expressions of SIRT1 and PGC-1α in the lung tissues were reduced in COPD rats compared to that in the control group, and were increased in the resveratrol intervention group compared with the COPD group (*p* < 0.05). Western blot analysis indicated that the protein expressions of SIRT1 (Figure 6A,B) and PGC-1α (Figure 6C,D) were increased in the resveratrol intervention group compared with the COPD group (*p* < 0.05). The real-time PCR detection results showed that in the COPD group SIRT1 and PGC-1α mRNA expression was significantly lower.

**Figure 7:**
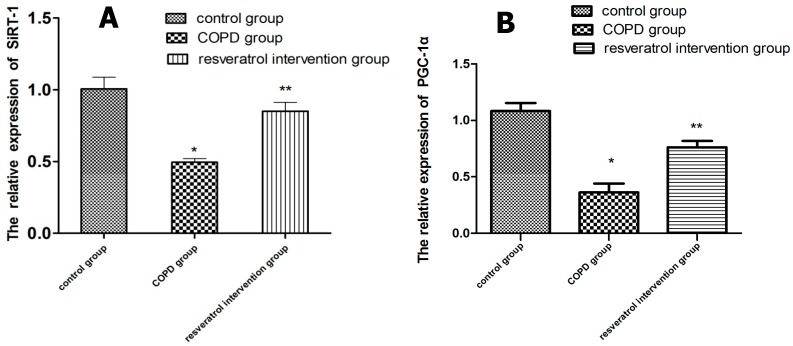
SIRT1 (**A**) and PGC-1α (**B**) mRNA expression in the lung of rats from the control (n = 10), COPD (n = 10) and resveratrol groups (n = 10). SIRT1 and PGC-1α mRNA expression measured by quantitative RT-PCR were decreased in lungs of COPD rats and increased by the resveratrol treatment. Resveratrol increased the expression of SIRT1 and PGC-1α in the lungs of rats with COPD. * *p* < 0.01, vs. Control group; ** *p* < 0.01, vs. COPD group.

SIRT1 and PGC-1α mRNA expression in the resveratrol group were increased compared with the COPD group (Figure 7) (*p* < 0.05).

## 3. Discussion

In the present study, we demonstrated that treatment with resveratrol attenuated CS- and LPS-induced lung inflammation in a rat model of COPD. Resveratrol administration significantly decreased levels of pro-inflammatory and oxidative stress [5]. In the study, the levels of pro-inflammatory cytokines such as IL-8 and IL-6 were increased after CS and LPS exposure. The resveratrol therapy significantly impeded the levels of these inflammatory cytokines (IL-8 and IL-6) and inflammatory cells in BALF, which may explain the anti-inflammatory activity of resveratrol. Resveratrol reduced MDA content and increased SOD, which may explain the anti-oxidative stress of resveratrol. For the first time, our results showed that SIRT1 and PGC-la expression were reduced by cigarette smoke and LPS, and elevated by resveratrol. These results imply that the effect of resveratrol on airway inflammation induced by cigarette smoke exposure might act through the SIRT1/PGC-lα pathways.

Inflammation and oxidative stress are thought to play a pivotal role in the pathogenesis of COPD [10]. The inflammatory environment in COPD leads to oxidative stress, as activated cells recruited to the airways produce excessive quantities of ROS [11]. IL-6 and IL-8 play key roles in the pathogenesis of stable and exacerbated COPD [12]. SOD is an anti-inflammatory enzyme as well as a major anti-oxidant [13] and MDA, a by-product of polyunsaturated fatty acid peroxidation, may be a reliable marker of oxidative stress in COPD [14]. Resveratrol has a cardiac protective effect against smoking and lipopolysaccharides (SM/LPS)-induced oxidative stress through upregulation of SOD activity [15]. Our results showed that treatment with resveratrol significantly improved the histological structure in the lung tissue. It also reduced in the extent of cellular infiltration, and the number of inflammatory cells. Our study also demonstrated that resveratrol significantly reduced pulmonary inflammation as determined by the numbers of total cells and the proportion of neutrophils in BALF. Resveratrol reduced the levels of IL-6 and IL-8, which may be potentially related to its anti-inflammatory property. We discovered that resveratrol has a protective effect through upregulation of SOD activity and regulation of MDA activity against CS/LPS induced oxidative stress.

SIRT1 has broad biological effects, which involve both oxidative stress and cell metabolism [16]. It has strong antioxidative stress and anti-apoptosis effects in the heart [17]. SIRT1, a longevity associated protein, is important in maintaining mitochondrial function [18]. Previous studies have demonstrated that the anti-inflammatory action of resveratrol may be mediated through enhancing SIRT1 expression [8]. A study demonstrated that IL-8 and IL-6 expressions were attenuated in cells pretreated with SIRT1 activators, and proinflammatory effects were exacerbated by the knockdown of SIRT1 expression [19]. Since resveratrol acts as a SIRT1 mimetic [15], we investigated the effects of resveratrol treatment on lung SIRT1 expression in COPD. The expression of SIRT1 was detected by western blotting and real-time PCR and immunohistochemical staining. Our results showed that SIRT1 was decreased in the lung of COPD rats and was reversed in COPD rats with resveratrol treatment. These findings suggest that resveratrol plays an important role in the regulation of inflammation and oxidants by SIRT1 in COPD.

More importantly, SIRT1 has been demonstrated to interact directly with PGC-1 to increase PGC-1α expression and mitochondria biogenesis [20]. SIRT1 was expressed in both the cytoplasm and nuclei in many metabolically active tissues, such as the PPAR-γ receptor and its coactivator PGC-1α [21]. PGC-1α suppresses ROS production in cells through the induction of ROS detoxifying enzymes. It has been demonstrated that decreased PGC-1α expression increases oxidative stress and neurodegeneration [22]. PGC-1α may not only stimulate mitochondrial biogenesis, but also protect neurons from oxidative injury through the induction of several ROS-detoxifying enzymes [23]. PGC-1α is also a master regulator of ROS-scavenging enzymes including Mn-SOD2, catalase and GSH-Px [24]. Resveratrol can stimulate SIRT1 and PGC-1α activation, which in turn may promote the expression of the slow, oxidative myogenic program in mdx mouse muscle [25]. ROS play important roles in COPD, which is regulated by inflammation mechanisms, and excessive ROS can directly initiate inflammatory responses [26]. PGC-1α null have reduced expression of the anti-ROS genetic program [22]. The SIRT1-PGC-1α signaling pathway can up-regulate the expression of antioxidant enzymes, scavenge free radicals, reduce intracellular oxidative stress levels, and reduce the degeneration of cells [27]. SIRT1/PGC-1α could be related to an ROS-initiated signaling cascade which requires further study in COPD. The present study revealed that the expression of PGC-1α was upregulated by the SIRT1 activator, resveratrol, in the COPD rats. The results suggested that the SIRT1/PGC-1α signaling pathway was inhibited in COPD. Further experiments found that, resveratrol significantly increased SOD and reduced MDA, enhanced the expression of PGC-1α mRNA and protein levels. SIRT1 could regulate transcription of PGC-lα expression. It was possible to enhance the activity of the SIRT1-PGC-lα signaling pathway, which could alter the oxidative stress reaction in COPD. Resveratrol was expected to become one of the new drugs in COPD.

Taken together, our results reveal that resveratrol has a therapeutic effect, which can regulate inflammation and oxidative stress via the activation of SIRT1 and their downstream targets, including PGC-1α in COPD rats. In COPD, more studies are still needed to confirm whether resveratrol has a lung protective effect due to the improvement of mitochondrial function through its activation of SIRT1/PGC-lα and then the reduction of oxidative stress. We will attempt to determine the exact mechanism in future work.

## 4. Materials and Methods

### 4.1. Animals and Cigarette Smoke Exposure

**Figure 8:**
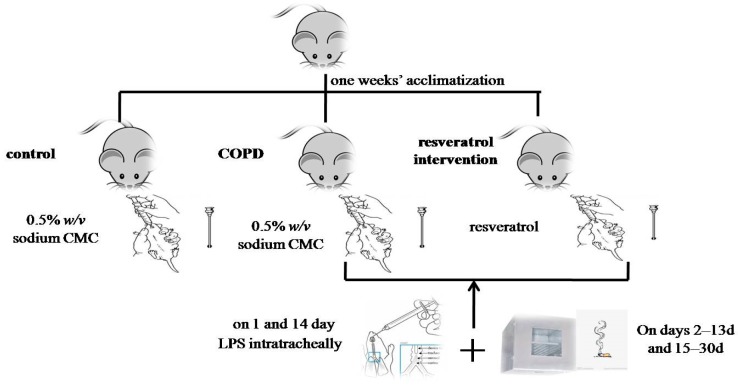
Schematic diagram of animal models.

This research was conducted according to internationally recognized guidelines on animal welfare and conducted in accordance with the guidelines of the Chinese Council on Animal Care and the experimental protocol was approved by Medical Ethics Committee of Jishou university (No. 2015021). Thirty Wistar male rats (250–300 g) (12–13 weeks) were obtained from the experimental animal center of technology services in Changsha, China. The Wistar rats were housed in temperature controlled room (25 ± 2 °C) with relative humidity (55 ± 10%) on a 12 h light/dark cycle during the study. After one weeks’ acclimatization to laboratory conditions, the Wistar rats were randomly divided into three groups as follows: (1) control group (n = 10) (2) COPD group (n = 10) (3) resveratrol intervention group (n = 10). Rats in the resveratrol intervention group were fed resveratrol by gavage device before smoking every day (50 mg/kg [28]) for 20 days. Rats in the COPD and control group were fed 0.5% *w*/*v* sodium Carboxy Methyl Cellulose(CMC) for 20 days. The COPD group and resveratrol intervention group were challenged with passive cigarette smoking and repeatedly instilled with 200 µg of LPS (Sigma-Alderich, St. Louis, MO, USA) intratracheally on the first day and on the 14th day, while the control rats were injected with 200 µL saline as previously described [29]. On days 2–13 days and 15–30 days of model establishment, the rats were placed in a self-produced fumigating box for smoking. The rats were exposed to the smoke of 15 cigarettes (Hongqi Canal^®^ Filter tip cigarette, Henan Tobacco Industry, Zhengzhou, China) for 20 min, twice daily, at an interval of 4 h except for one and 14 days [30] (Figure 8). The tar content was 17 mg per cigarette and the concentration of smog was about 18% (*v*/*v*) within the box with five cigarettes burning concurrently. The control group was not given fumigation.

Resveratrol was purchased from Sigma (Sigma, St. Louis, MO, USA) and suspended in 0.5% *w*/*v* sodium CMC, and administered 1 h before the cigarette smoke exposure or LPS instillation. 

### 4.2. Lung Histological Examination

The inferior lobes of the right lung tissues were fixed with 4% formaldehyde phosphate buffer overnight and then dehydrated and paraffin embedded and sliced into 4 µm sections and stained with hematoxylin and eosin. The slides were observed under a Leica photograph microscope (Leica Microscope Ltd., Wetzlar, Germany) at 200× magnification to evaluate the morphological changes in the lungs.

### 4.3. Serum Collection

Thirty days after exposure to cigarette smoke, the rats were anesthetized by pentobarbital sodium. After anesthesia, blood samples were collected from the common carotid artery intubation by tube, and then centrifuged at 3500 r/min for 10 min. The serum supernatant fluids were taken into tubes and taken into a −80 °C low temperature refrigerator.

### 4.4. Preparation of BALF and Tissue Processing

Thirty days after the exposure to cigarette smoke, the day after collecting the blood samples, the right side of the main bronchus was tied. The left lungs were lavaged three times with a 3 mL saline solution warmed at 37 °C via the tracheal cannula, and the BALF was collected. All BALF samples were immediately centrifuged at 1200 rpm for 10 min at 4 °C. The supernatants were obtained and stored at −80 °C for further analysis. The cells were resuspended in a phosphate-buffered saline (PBS) solution (300 μL) and counted via a hemocytometer. The cell differential was determined from an aliquot of the cell suspension (100 μL) by centrifugation on a slide and Wright-Giemsa stain. A total of 200 leukocytes were counted in each BALF sample, and the percentage of neutrophils was calculated based on morphological criteria.

The right middle lobes of the lung tissues were homogenized in potassium phosphate buffer (pH 7.4) and centrifuged at 4800 rpm for 30 min at 4 °C; the supernatants were employed for the analysis of SOD and MDA. The inferior lobes of right lung tissues were for the histopathological examination and immunohistochemical assay. The upper lobes of the right lung tissues were perfused with ice-cold heparinized saline, isolated and stored at −80 °C until analysis. The upper lobes of the right lung tissues were used for western blotting and real-time PCR.

### 4.5. Determination of Levels of Inflammation 

The levels of IL-6 and IL-8 in the serum were measured with the ELISA method using commercially available kits (R&D Systems, Minneapolis, MN, USA). Mouse IL-8 and IL-6 assay kits were purchased from Wuhan Boster Bio-engineering limited company. The procurement operation was performed according to the manufacturer’s instructions. The serum samples were added to 96-well microtiter plates and incubated for 30 min at 37 °C with anti-rat IL-6 and IL-8 in a coating buffer at a dilution of 1:100. Then, the wells were washed five times with PBS before adding peroxidase-labeled biotinylated secondary antibodies. After 30 min of induction at 37 °C, the plates were treated with a tetramethylbenzidine (TMB) substrate solution for 10 min, and the reaction was stopped by the addition of a TMB stop solution. Finally, the optical density (OD) was measured at 450 nm by a microplate reader (1510, Thermo Fisher Scientific, Vantaa, Finland). All specimens were tested at the same time.

### 4.6. Determination of Levels of Oxidative Stress

In order to determine the antioxidant defenses, we measured the enzymatic defense activities in the BALF and lung tissues [31]. The SOD and MDA in both BALF and pulmonary tissue homogenate were measured in this research. The levels of MDA and SOD in BALF and tissue homogenates were determined using the MDA and SOD kits (Nanjing Jiancheng Bioengineering Institute, China), with xanthinoxidase (SOD) and thiobarbituric acid (TBA) (MDA) chromometry, with enzyme-labelled meters at the 550 nm (SOD) and 532 nm (MDA) wave lengths, according to the manufacturer’s instructions. The homogenate supernatant and the BALF were used on the same day for the assay of the oxidative biomarkers MDA and SOD. 

### 4.7. Immunohistochemical

The immunohistochemical study of SIRT1 and PGC-1α were performed on formalin-fixed, paraffin-embedded tissue sections obtained from the rats with the inferior lobes of right lung tissues. Paraffin-embedded 4 μm thick serial sections were subjected to paraffin removal and rehydrated through graded alcohol. To block the endogenous peroxidase activity, the slides were pretreated with 3% H_2_O_2_. Tissue sections were boiled in a 0.01 M sodium citrate buffer (pH 6.0) in a 1000-watt microwave oven for 10 min to retrieve cell antigens. Primary antibodies were diluted to 1:100 for rabbit anti-SIRT1 and rabbit anti-PGC-1α. The sections were incubated with the primary antibody at 4 °C overnight. Subsequently, the slides were incubated with goat anti-rabbit biotinylated secondary antibody at a concentration of 1:100 for 30 min at 37 °C and then reacted with streptavidin-peroxidase conjugate for 30 min at 37 °C. After several further washes with phosphate buffer, slides were treated with diaminobenzidine (DAB) and counterstained with hematoxylin. The sections were dehydrated, mounted and observed under a light microscope. Omitting the primary antibody for each protein was used as the negative control, and the sections did not show any background staining.

### 4.8. Real-Time PCR 

For total RNA extraction, the upper lobes of the right lung tissues (100 mg) were dissolved in Trizol (1 mL) (Life Technologies, Grand Island, NY, USA). RNA was reverse transcribed by use of Moloney Murine Leukemia Virus Reverse (M-MLV) reverse transcriptase (Fermentas, Glen Burnie, MD, USA). An ABI 7500 real-time thermocycler (Applied Biosystems, Foster City, CA, USA) was used to monitor the amplification reactions in real time. The initial activation was at 95 °C for 10 s, 60 °C for 30 s, and 40 cycles; and 95 °C for 15 s, 60 °C for 1 min, 95 °C for 15 s, and 60 °C for 30 s (ABI, Foster City, CA, USA). Real-time PCR was performed using theLightCycler1.5 (Applied Biosystems, Carlsbad, SC, USA) and the SYBRGreenqRCR Mix (Takara, Otsu, Japan).The mouse SIRT1 and PGC-1α primers used for the PCR were as follows: 5′-TGTGGTGAAGATCTATGGAGGC-3′ (forward) and 5′-TGTACTTGCTGCAGACGTGGTA-3′ (reverse) for SIRT1 and 5′-TATGGAGT GACATAGAGTGT GCT-3′ (forward) and 5′-GTCGCTACACCACTTCAATCC (reverse) for PGC-1α and 5′-CCAAGGCCAACCGCGAGAAGATGAC (forward) and 5′-AGGGTACATGGTGGTGCCGC CAGAC (reverse) for β-action. The values of the target genes were normalized using the value of the housekeeping gene β-action. All samples were run in triplicate and the average values were calculated. Omitting cDNA was used as the negative control.

### 4.9. Western Blotting of SIRT1 and PGC-1α

The upper lobes of the right lung tissues (100 mg) were homogenized inRadio Immunoprecipitation Assay (RIPA ) lysis buffer (50 mM Tris, pH 7.4, 150 mM NaCl, 0.1% SDS, 0.25% sodium deoxycholate, 2% Triton-X100, 1 mM PMSF, 2 µg/mL leupeptin) supplemented with appropriate protease inhibitors (Auragene, Changsha, China). Soluble proteins were recovered after centrifugation at 13,000 rpm for 20 min at 4 °C. Subsequently, the protein concentration was measured with the Bio-Rad protein assay kit (Bio-Rad Laboratories, Hercules, CA, USA) spectrophotometrically at 570 nm. The sample was incubated at 100 °C for 20 min. An 8% SDS-polyacrylamide gel electrophoresis (SDS-PAGE) gel was run at 200 V for 45 min. The proteins were transferred to a polyvinylidene fluoride (PVDF) membrane and were blocked with 5% nonfat dry milk in 0.05% TBS-Tween-20 for 1 h at room temperature. Then blots were incubated with rabbit anti-SIRT1 (110 kDa) (1:1000 dilution, Proteintech, Wuhan, China) and rabbit anti-PGC-1α (91 kDa) (1:1000 dilution, Proteintech, Wuhan, China) and β-actin (1:2000 dilution, Proteintech, Wuhan, China) antibodies in blocking buffer overnight at 4 °C. After washing with 0.5% TBS-Tween three times, membranes were incubated with horseradish peroxidase-conjugated goat anti-rabbit IgG antibody (1:18,000 dilutions, Auragene, Changsha, China) for 1 h at room temperature. Proteins were detected with enhanced luminol-based chemiluminescent (ECL) reagents (Amersham Pharmacia Biotech, Basel, Switzerland). Densitometry evaluation was performed using Quantity One software (Bio-Rad Laboratories, Hercules, CA, USA).

### 4.10. Statistical Analysis

Data is presented as mean ± SEM (n = 10). For the purpose of multiple comparison, one way analysis of variance (ANOVA) followed by Bonferroni’s multiple comparison test were applied using the biostatistics software SPSS15.0. (International Business Machines Corporation, Palo Alto, SC, USA) Significance was assigned at *p* < 0.05.

## 5. Conclusions

In conclusion, this study suggests that resveratrol is effective in preventing lung inflammatory response and oxidative stress in a rat model of COPD. Our findings provide the rationale for further studies directed towards understanding of mechanism of resveratrol in preventing inflammation.

## References

[1] Shergis J.L., Di Y.M., Zhang A.L., Vlahos R., Helliwell R., Ye J.M., Xue C.C. (2014). Therapeutic potential of Panax ginsengand ginsenosides in the treatment of chronic obstructive pulmonary disease. Complement. Ther. Med..

[2] Fischer B.M., Pavlisko E., Voynow J.A. (2011). Pathogenic triad in COPD: Oxidativestress, protease–antiprotease imbalance, and inflammation. Int. J. Chron. Obstruct. Pulmon. Dis..

[3] Herr C., Han G., Li D., Tschernig T., Dinh Q.T., Beißwenger C., Bals R. (2015). Combined exposure to bacteria and cigarette smoke resembles characteristic phenotypes of human COPD in a murine disease model. Exp. Toxicol. Pathol..

[4] Chen J., Zhou H., Wang J., Zhang B., Liu F., Huang J., Li J., Lin J., Bai J., Liu R. (2015). Therapeutic effects of resveratrol in a mouse model of HDM-induced allergic asthma. Int. Immunopharmacol..

[5] Milevoj K.L., Domijan A.M., Posavac K., Čepelak I., Žanić G.T., Rumora L. (2016). Systemic redox imbalance in stable chronic obstructive pulmonary disease. Biomarkers.

[6] Rahman I., Kinnula V.L., Gorbunova V., Yao H. (2012). SIRT1 as a therapeutic target in inflammaging of the pulmonary disease. Prev. Med..

[7] Lavu S., Boss O., Elliott P.J., Lambert P.D. (2008). Sirtuins—Novel therapeutic targets to treat age-associated diseases. Nat. Rev. Drug Discov..

[8] Said R.S., E-Demerdash E., Nada A.S., Kamal M.M. (2016). Resveratrol inhibits inflammatory signaling implicated in ionizing radiation-induced premature ovarian failure through antagonistic crosstalk between silencing information regulator 1 (SIRT1) and poly (ADP-ribose) polymerase 1 (PARP-1). Biochem. Pharmacol..

[9] Knobloch J., Wahl C., Feldmann M., Jungck D., Strauch J., Stoelben E., Koch A. (2014). Resveratrol attenuates the release of inflammatory cytokines from human bronchial smooth muscle cells exposed to lipoteichoic acid in chronic obstructive pulmonary disease. Basic Clin. Pharmacol. Toxicol..

[10] Tuder R.M., Petrache I. (2012). Pathogenesis of chronic obstructive pulmonary disease. J. Clin. Investig..

[11] Wood L.G., Wark P.A., Garg M.L. (2010). Antioxidant and anti-inflammatory effects of resveratrol in airway disease. Antioxid. Redox Signal..

[12] Knobloch J., Sibbing B., Jungck D., Lin Y., Urban K., Stoelben E., Strauch J., Koch A. (2010). Resveratrol impairs the release of steroid-resistant inflammatory cytokines from human airway smooth muscle cells in chronic obstructive pulmonary disease. J. Pharmacol. Exp. Ther..

[13] Matés J.M. (2000). Effects of antioxidant enzymes in the molecular control of reactive oxygen species toxicology. Toxicology.

[14] Antus B., Harnasi G., Drozdovszky O., Barta I. (2014). Monitoring oxidative stress during chronic obstructive pulmonary disease exacerbations using malondialdehyde. Respirology.

[15] Hu Y.X., Cui H., Fan L., Pan X.J., Wu J.H., Shi S.Z., Cui S.Y., Wei Z.M., Liu L. (2013). Resveratrol attenuates left ventricular remodeling in old rats with COPD induced by cigarette smoke exposure and LPS instillation. Can. J. Physiol. Pharmacol..

[16] Chong Z.Z., Shang Y.C., Wang S., Maiese K. (2012). SIRT1: New avenues of discovery for disorders of oxidative stress. Expert. Opin. Ther. Targets.

[17] Hsu C.P., Odewale I., Alcendor R.R., Sadoshima J. (2008). SIRT1 protects the heart from aging and stress. Biol. Chem..

[18] Fusco S., Maulucci G., Pani G. (2012). Sirt1: Def-eatingsenescence?. Cell Cycle.

[19] Cao L., Liu C., Wang F., Wang H. (2013). SIRT1 negatively regulates amyloid-beta-induced inflammation via the NF-κB pathway. Braz. J. Med. Biol. Res..

[20] Nemoto S., Fergusson M.M., Finkel T. (2005). SIRT1 functionally interacts with the metabolic regulator and transcriptional coactivator PGC-1α. J. Biol. Chem..

[21] Nicoletti N.F., Rodrigues-Junior V., Santos A.A., Leite C.E., Dias A.C., Batista E.L., Basso L.A., Campos M.M., Santos D.S., Souto A.A. (2014). Protective effects of resveratrol on hepatotoxicity induced by isoniazid and rifampicin via SIRT1 modulation. J. Nat. Prod..

[22] St-Pierre J., Drori S., Uldry M., Silvaggi J.M., Rhee J., Jager S., Handschin C., Zheng K., Lin J., Yang W. (2006). Suppression of reactive oxygen species and neurodegeneration by the PGC-1 transcriptional coactivators. Cell.

[23] Chen S.D., Lin T.K., Yang D.I., Lee S.Y., Shaw F.Z., Liou C.W., Chuang Y.C. (2010). Protective effects of peroxisome proliferator-activated receptors gamma coactivator-1α against neuronal cell death in the hippocampal CA1 subfield after transient global ischemia. J. Neurosci. Res..

[24] Chen S.D., Yang D.I., Lin T.K., Shaw F.Z., Liou C.W., Chuang Y.C. (2011). Roles of oxidative stress, apoptosis, PGC-1α and mitochondrial biogenesisin cerebral ischemia. Int. J. Mol. Sci..

[25] Vladimir L., Matthew B., John A.L., Bernard J.J. (2014). Resveratrol induces expression of the slow, oxidative phenotype in mdxmouse muscle together with enhanced activity of the SIRT1-PGC-1α axis. Am. J. Physiol. Cell Physiol..

[26] Zuo L., Lucas K., Fortuna C.A., Chuang C.C., Best T.M. (2015). Molecular Regulation of Toll-like Receptors in Asthma and COPD. Front. Physiol..

[27] Lagouge M., Argmann C., Gerhart-Hines Z., Meziane H., Lerin C., Daussin F., Messadeq N., Milne J., Lambert P., Elliott P. (2006). Resveratrol improves mitochondrial function and protects against metabolic disease by activating SIRT1 and PGC-1α. Cell.

[28] Sadarani B.N., Majumdar A.S. (2015). Resveratrol potentiates the effect of examethasone in rat model of acute lung inflammation. Int. Immunopharmacol..

[29] Nie Y.C., Wu H., Li P.B., Luo Y.L., Zhang C.C., Shen J.G., Su W.W. (2012). Characteristic comparison of three ratmodels induced by cigarette smoke or combined with LPS: To establish a suitable model for study of airway mucus hypersecretion in chronic obstructive pulmonary disease. Pulm. Pharmacol. Ther..

[30] Tang W., Xie J., Xu S., Lv H., Lin M., Yuan S., Bai J., Hou Q., Yu S. (2014). Novel nitric oxide-releasing derivatives of brusatol as antiinflammatory agents: Design, synthesis, biological evaluation, and nitric oxide release studies. J. Med. Chem..

[31] Da Cunha A.A., Nunes F.B., Lunardelli A., Pauli V., Amaral R.H., de Oliveira L.M., Saciura V.C., da Silva G.L., Simoes Pires M.G., Fagundes Donadio M.V. (2011). Treatment with *N*-methyl-D-aspartate receptor antagonist (MK-801) protects against oxidative stress in lipopolysaccharide-induced acute lung injury in the rat. Int. Immunopharmacol..

